# Autopsy and its role in Franco’s dictatorship: a case of the last Republican mayor of the town *Calera y Chozas* (Toledo, Spain)

**DOI:** 10.1007/s12024-022-00497-y

**Published:** 2022-07-25

**Authors:** Nataša Šarkić, Miriam Saqqa Carazo, Lucia Muñoz Ugarte, Jesús Herrerín López

**Affiliations:** 1 Bioarchaeological Company AITA Bioarch, Barcelona, Spain; 2 Spanish National Research Council, Madrid (CSIC)/Universidad Complutense of Madrid (UCM), Madrid, Spain; 3 Madrid, Spain; 4 Dpto. Ciencias de la Vida. Facultad de Ciencias, Universidad de Alcalá, Crta. Madrid-Barcelona, km 3,6. 28850 Alcalá de Henares, 28049 Madrid, Spain

**Keywords:** Spanish Civil War, Exhumation, Physical anthropology, Mass grave, Identification, Head injuries, Autopsy

## Abstract

In the town of Calera y Chozas (Spain), five mass graves containing the remains of 28 individuals were discovered during a 2012 excavation. The witnesses and historical evidence indicated that the body of the last Republican mayor of the town, Felipe Fernández Varela, who had died in September 1939, was located in the mass grave designated as no. 1. Within this particular grave, only two bodies were found. Anthropological analysis showed that the first individual was significantly younger than 50 years, being the mayor’s age at the time of death, while the age of the second individual was closer to 50. This second individual had a fractured skull, with a depression on the left parietal bone, and there were unmistakable signs of autopsy, which consisted of cut marks on the frontal bone and the sternal extremity of the right clavicle. Further historical research revealed documents concerning the autopsy performed on this individual. Although, according to the report, the cause of death was a stroke — the consequence of atherosclerosis and alcoholism — no reference was made to the forceful impact to the skull or intracranial bleeding. Considering the size of the fracture on the skull and the fact that there were no signs of bone healing, we believe that this impact, and not the stroke, was the direct cause of the death of the last Republican mayor. The mayor’s case is a clear example of the role forensic medicine performed at the beginning of Franco’s dictatorship. The task was not only to conceal the crime but also to tarnish the victim’s name.

## Introduction

The rebel army’s *coup d’etat* against the legitimate government of the Spanish Republic led to a 3-year military conflict known as the Spanish Civil War (1936–1939). The army and its related militias (monarchists, conservative Catholics, and Falangists) systematically eliminated political opposition during the war and the subsequent dictatorship of Francisco Franco. Historians estimate that during the war and the first years of the dictatorship, between 130,000 and 150,000 people were victims of extrajudicial executions and death sentences from court-martial and popular tribunals [1]. Authorities buried the victims of these political executions in mass graves which were frequently placed at a distance from villages, along roads, or at the edges of cemeteries [2].

Since 2000, at the request of the families of victims of fascist violence, officials have exhumed at least 740 mass graves and 9,000 remains of people killed during and after the war [3]. These forensic exhumations serve not only to recover and identify the victims’ remains, but also to shed light on the processes of violence exerted and allow a greater understanding of the structures of state repression.

**Fig. 1:**
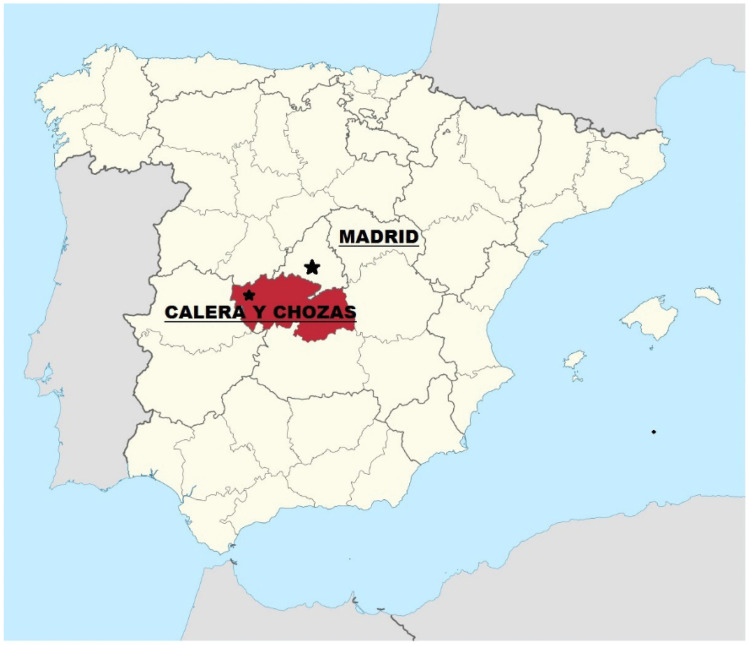
Map of Spain with the location of the village Calera y Chozas

In the town of Calera y Chozas, Toledo (Fig. 1), five mass graves were excavated [2]. The excavation was requested by relatives of victims killed during the civil war. According to various sources and oral testimony from both locals and victims’ relatives, the mass graves had been dug a few meters south of the current cemetery [2]. According to this information, the group “Memoria Histórica de Calera y Chozas” began prospecting for mass graves that might contain the remains of missing relatives, killed between 1936 and September 1939.

A total of five mass graves were uncovered during the summer of 2012. Twenty-eight individuals were recovered, two of whom were women. The ages of the victims ranged from 13 to 60 + .

According to the historical evidence and the testimonies collected by archeologists, it was acknowledged that the last Republican mayor of the town, Felipe Fernández Varela, who died in September 1939, was buried in grave no. 1. This grave had only 2 occupants — a well-preserved male individual approximately 35–40 years old, and another of 45–50 years. Therefore, the evidence suggested that the mayor was more likely to correspond to the second individual, named CAL-02, as we knew that he was in his fifties at the time of his death.

## Case report

Human skeletal remains of CAL-02 were cleaned and analyzed in the laboratory of Physical Anthropology at the Autonomous University of Madrid. The preservation of this individual was excellent, with Preservation Index = 100%, meaning all the bones were preserved [4, 5]. In order to determine the sex, the morphological characteristics of the coxal, using as the reference methods of Ferembach et al. [6] and Bruzek [7], were considered, initially. Secondly, we studied the cranial and mandibular morphology, using the methods of Herrmann et al. [8], Acsadi and Nemeskeri [9], Buikstra and Ubelaker [10], Cuijpers et al. [11], and Loth and Henneberg [12].

To determine the age at the time of death, we used a set of methods applicable to different areas of the skeleton: changes on the pubic symphyseal surface [13], auricular surface of the ilium [14], changes in the fourth rib on the articular surface with the sternum [15], and the wearing down of the occlusal surface of the tooth [16, 17]. His stature was slightly above average for the male population of Calera and Chozas, which was 165.10 cm, according to the method of Pearson [18], and 163.45 cm, according to the Mendonça method [19].

**Fig. 2:**
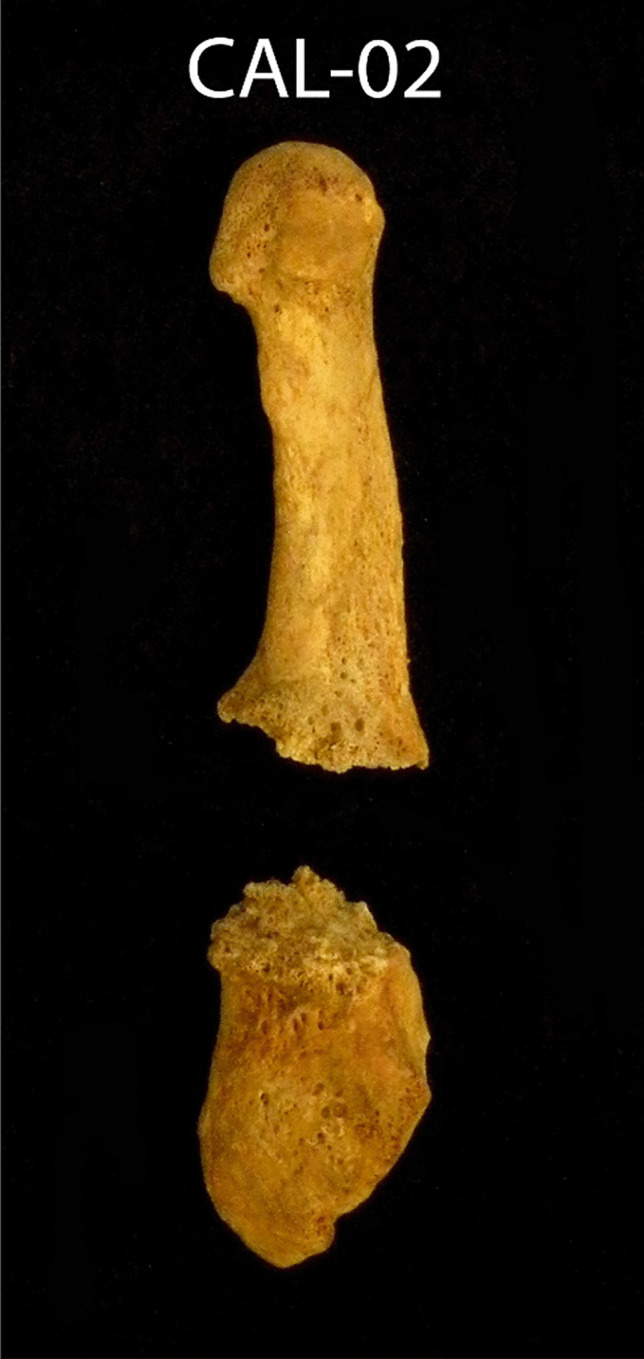
A transverse fracture perimortem on the fifth metatarsal of the left foot

In order to identify the causes of his death, the following information analyzes the traumatic injuries: a transverse fracture was noted on the fifth metatarsal of the left foot (Fig. 2). The fracture line is straight and the bone is clearly divided. The fracture did not occur immediately before death, as evidenced by the bone reaction on both sides of the fracture. However, it did not occur very long before the individual’s death since there had been no fusion or callus formation.

**Fig. 3:**
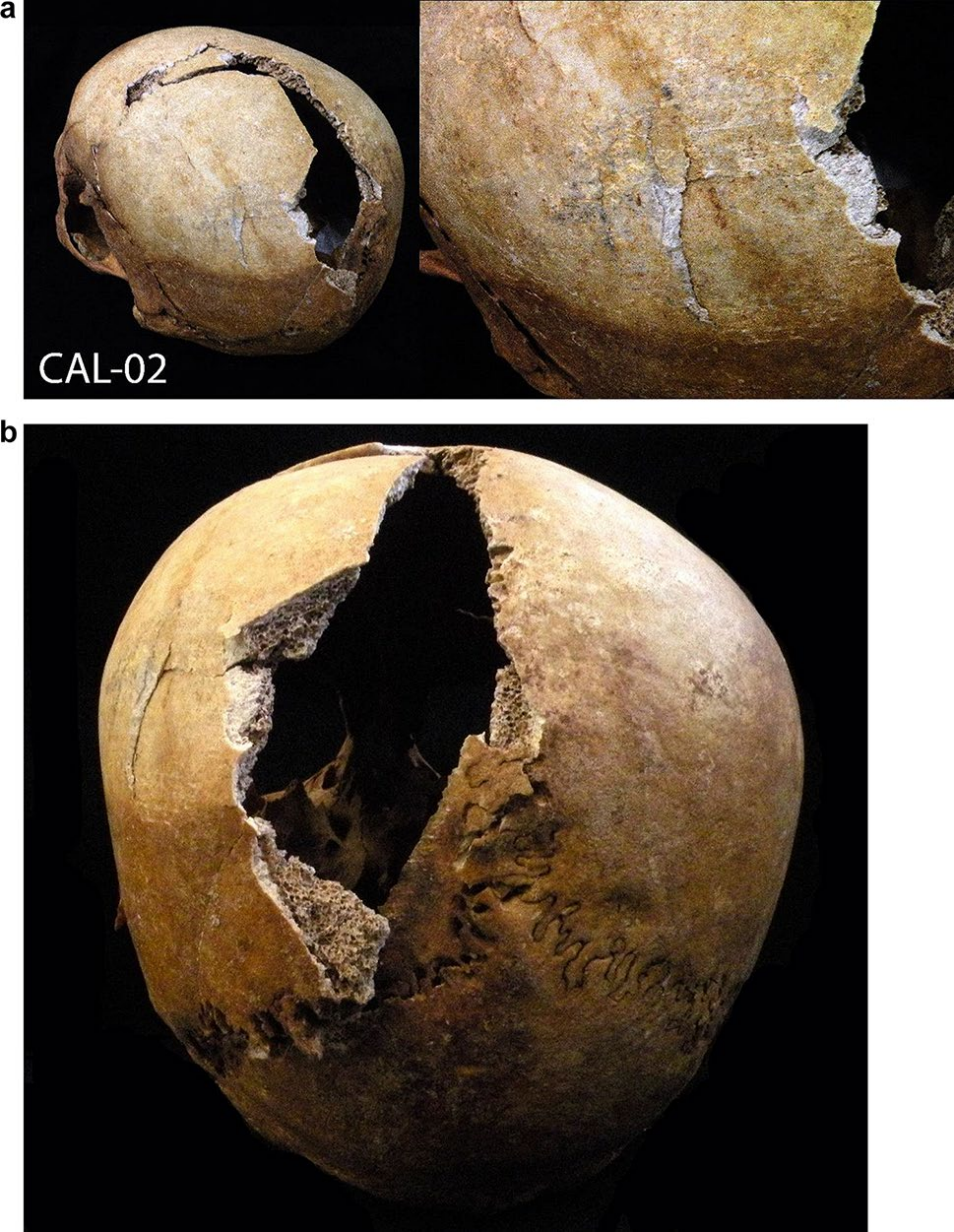
**a**, **b** A strong impact observed on the left parietal lobe, possibly the consequence of a hit with a blunt object or a fall on the floor

On the left parietal lobe, traces of a forceful impact are observed, possibly the consequence of a blunt force trauma or a fall (Fig. 3a, b). The postmortem fracture near the sagittal suture does not allow us to fully observe whether it was a fracture of the occiput but it is clear that a strong impact caused the depressed skull fracture of the external table. No bone reaction or fusion was noted.

**Fig. 4:**
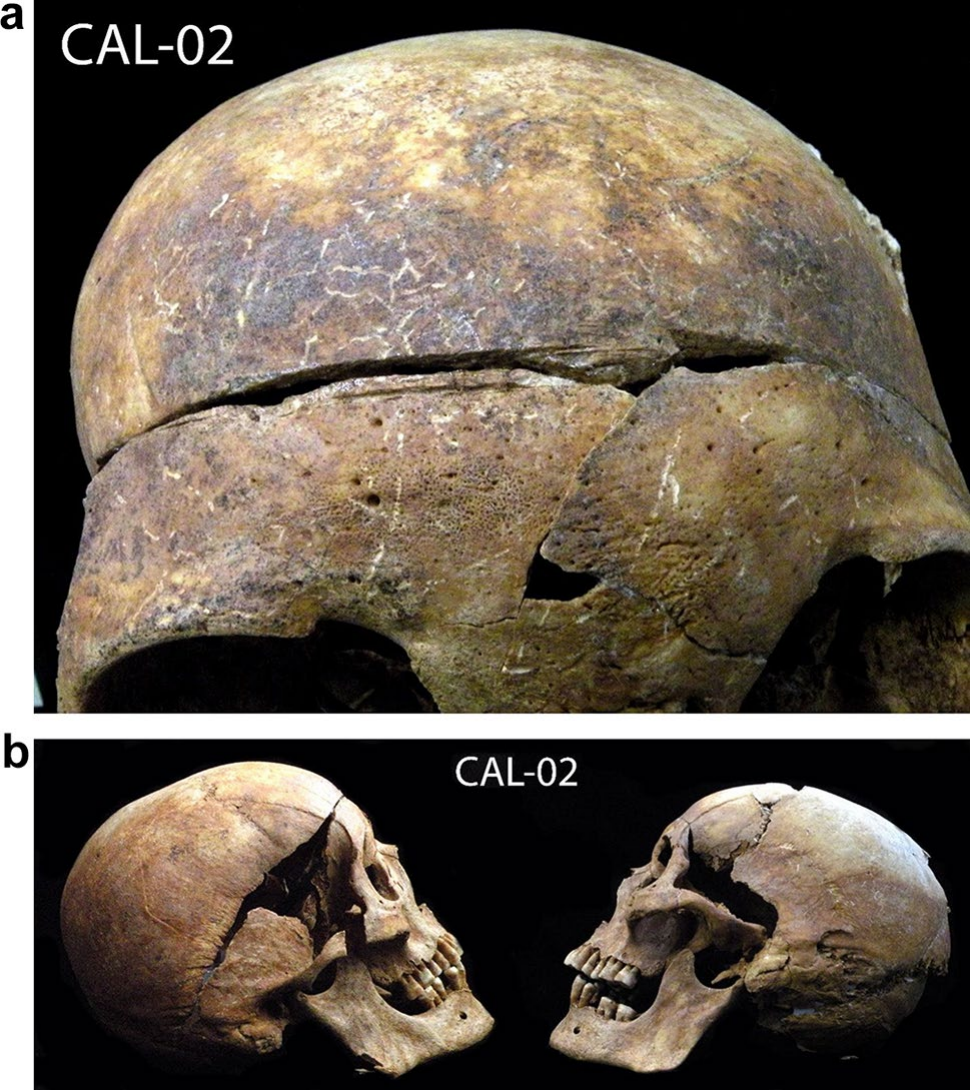
**a**, **b** A straight, parallel cut marks, very like those made with medical instruments in autopsies, starting from the frontal lobe and ending on the right parietal bone

**Fig. 5:**
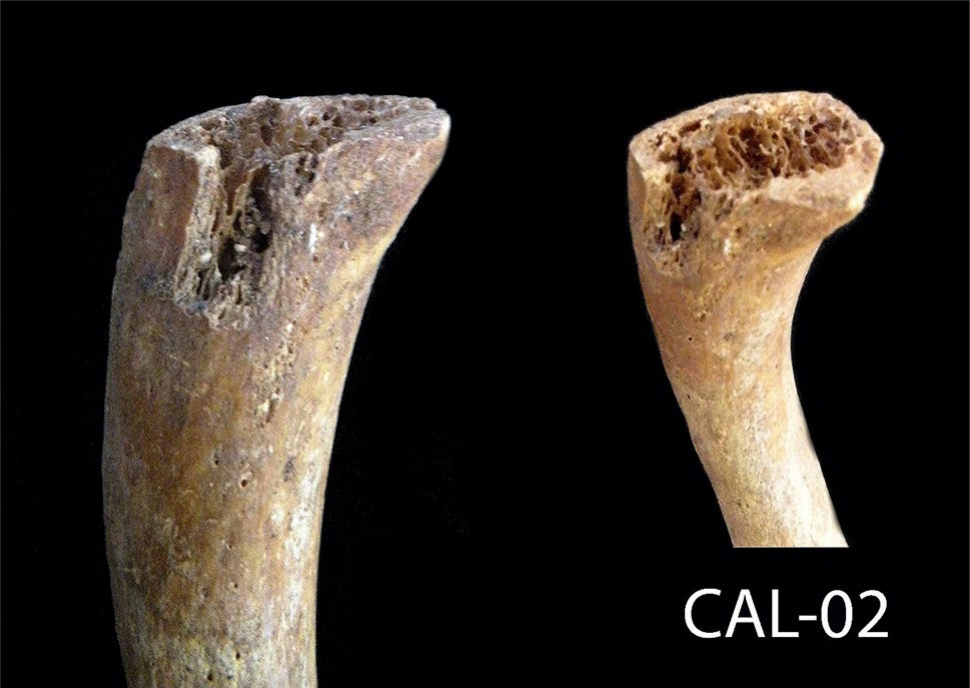
A straight, precise, vertical cut, very like those made with medical instruments in autopsies, noted on the right clavicle

Precise, linear, parallel cut marks on the skull were observed, starting from the frontal lobe, and ending on the right parietal bone (Fig. 4a, b). We also found a linear, precise, vertical cut, on the right clavicle, similar to those made with medical instruments in autopsies (Fig. 5).

## Discussion

Various traumatic injuries were noted on this individual, but they originated from different periods. The fracture on the fifth metatarsal had a bone reaction on its surface, but without forming a callus; therefore, it most likely occurred several weeks before the CAL-02 death. A blow to the parietal bone was *perimortem*, and no reaction on the bone was noted. The impact must have been of great intensity. Cranial high-energy fractures, as in this case, have a very high risk of intracranial hematoma. If subdural bleeding is significant, it can cause compression of the brain and possibly death [20].

Linear cuts found on the frontal bone are very straight and clearly made by a tool that was perpendicular to the surface, while the individual was in the supine position. Therefore, it cannot be a consequence of interpersonal violence and can only be explained by autopsy. Saw marks can be seen on the frontal bone, while the rest of the cut marks are not visible due to the fragmented condition of the temporal bones (Fig. 7). However, the saw marks do not occur on the posterior part of the skull, suggesting that the skull was only partially open, without removing the brain. The surgical cuts on the right clavicle, which are too vertical and well defined to be a consequence of interpersonal violence, could be attributed to an autopsy. Moreover, it was common forensic practice at the time to remove the clavicle’s mid-shaft to facilitate the investigation of the chest cavity, specifically for the removal of the heart and organs of the neck [23, 24]. Traces of those incisions were not noted on the ribs, probably due to the poor state of preservation of the ribs which prevented us from observing the possible presence of cuts on the ribs.

Unlike the vast majority of the individuals from the mass graves of Calera and Chozas, the skull of CAL-02 has no bullet hole, which indicates that he could have died from the impact received to the head. Later, after performing the autopsy, his corpse was taken and buried with other victims.

**Fig. 6:**
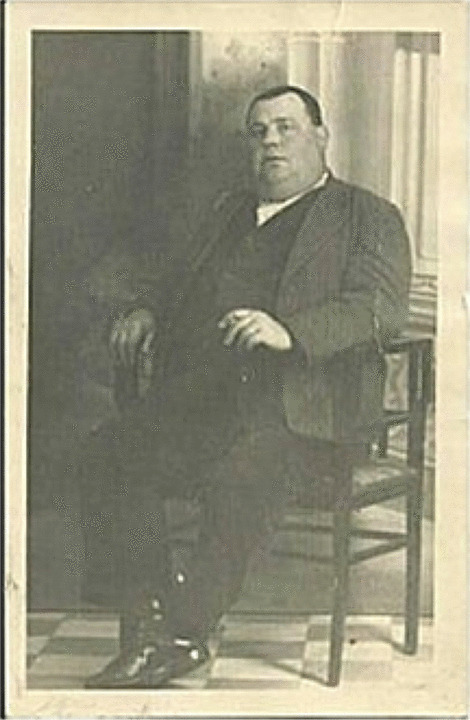
Felipe Fernández Varela, mayor of Calera y Chozas

As previously mentioned, through interviews with people from the village, and above all for the consistent historical research [20], we knew that in grave 1, one of the buried individuals was the last Republican mayor of the town and president of the revolutionary committee, Felipe Fernández Varela. Due to lack of funds, we could not perform DNA testing for maximum reliability of identification, but all the personal characteristics of the mayor (he was an obese person (Fig. 6), 50 years old, close to 1.65 m height) and along with autopsy evidence, consistent with the historical forensic records located, fit the results we obtained from analyzing CAL-02. Therefore, the identification in this case was done using historical evidence, testimonies, forensic archives, and anthropological analyses following the *Protocol for action in exhumations of victims of the civil war and dictatorship* published on 26 April 2011 in the Official State Gazette, by the Spanish government.^1^ The lack of DNA analysis cannot be an obstacle for obtaining a valid identification of the victims buried in Spanish mass graves. In effect, only 0.2% of the 130,199 estimated murder victims have been identified through DNA in the last 20 years [22].

Witness testimony confirmed that Felipe was arrested, taken to the prison, and subsequently interrogated, beaten, and tortured over approximately 2 weeks [21]. On 2nd September 1939, the date on which three years of the “liberation” of Calera by the National army troops was celebrated, Fernández was taken from the authorized prison, where he had been tortured [21]. According to oral testimonies, he was taken to the balcony of the Town Hall and thrown headfirst into the public square [21]. Eventually, he lost consciousness and was later taken to jail, where he died.

Considering all the physical characteristics and the impact on the skull, we concluded that this individual, CAL-02, was the last Republican mayor. A not consolidated fracture of the fifth metatarsal also fit the profile — it could have been a consequence of torture in prison. Nevertheless, one thing remained unclear: if this person was Felipe, who was killed as an enemy of Franco’s regime, what purpose did the autopsy serve?

After the completion and submission of the reports to the association of relatives of the exhumed victims [25], we were informed that the Civil War investigator from the University of Jerusalem, Javier de la Puerta, possessed a copy of the forensic report of the autopsy performed on the last Republican mayor of Calera y Chozas, referred to as CAL-02.

**Fig. 7:**
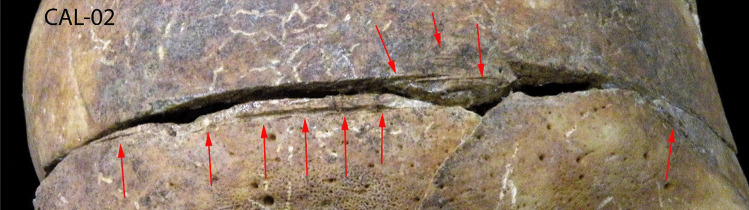
Linear cuts, made by a saw, were found on the frontal bone, proving that a brain autopsy was performed (red arrows)

Documents were forwarded to us, which confirmed the death of Felipe in the town jail and subsequent analysis of the corpse carried out by two local doctors. The autopsy report mentions “opening the cranial cavity, observing the heavily congested brain tissue, a small amount of blood flowing,” but says nothing about the trauma to the parietal bone, merely indicating that “light abrasions can be seen on the face and two small contusions on the head”^2^ (Fig. 7). According to this report, the cause of death was stroke — a consequence of atherosclerosis and alcoholism. In the report, “heart with big quantity of fat” was also mentioned.

When confronted by the data obtained through the anthropological study, the validity of the autopsy performed on the last Republican mayor of Calera y Chozas seems highly dubious. Forensic processes were subject to the political and social power structures of the time and the link between the discipline of forensic medicine and repressive regimes is of both historical and anthropological interest.

Similar cases of autopsy were found during the exhumation of Carcavilla (Palencia), a mass grave in the old city cemetery, where a total of 348 individuals were buried [26]. In this instance, the forensic practices made an intentional distinction between autopsy procedures carried out on people sentenced to extrajudicial death as a form of retaliation, which present more detailed autopsy findings, with the forensic description of the injuries that caused the death. In contrast, the cases of executions after military trials were issued with brief and imprecise death certificates in relation to the causes of death. An interesting example is the one in which the autopsy gives details where it indicates “(…) wound entry hole of a firearm projectile (…) another entrance hole in the right iliac fossa, at McBurney’s point”; but on the death certificate of the same individual, it only indicates the cause of death as “a consequence of an injury to the lung and cecum with hemorrhage in the hemithorax and abdomen,” without mentioning that this injury was caused by a firearm projectile [26].

Another example of forensic practices implemented during Francoist repression would be the investigation carried out in Valdenoceda prison (1938–1943). The interdisciplinary investigation included anthropological analysis of the remains found in the prison cemetery, together with the analysis of civil, forensic, and religious archives, which revealed the existence of several autopsy cases performed on people who died while incarcerated [27]. This study showed inconsistencies between the causes of death indicated by the autopsy and the information obtained by anthropological analysis [27]. In several cases, the anthropological analysis confirmed the presence of traumatic perimortem injuries on the skeletons, although these injuries were not mentioned in the forensic reports [27], as in the case of CAL-02. The same study also highlighted two cases where the autopsy reports determined the cause of death as “suicide,” but the oral testimonies of the time indicated that the victims were beaten to death by prison guards [27]. Once more, one can draw parallels with the case of the mayor of Calera y Chozas. The victim was thrown headfirst from a balcony; however, the medics who carried out the autopsy reported the cause of death as a stroke.

## Conclusion

In 2012, the research team “Memoria Histórica de Calera y Chozas” excavated five mass graves containing the remains of their missing relatives, killed between 1936 and September 1939. One of them was the last Republican mayor, Felipe Fernández Varela, who died in September 1939.

After an anthropological analysis, it was found that the individual CAL-02 corresponds to the mayor in terms of his age and physical characteristics. This was a male individual, slightly taller than average for that population, 45–50 years old, and overweight. Unlike most of the other exhumed individuals, CAL-02 had no evidence of gunshot wounds on the skeleton. He had a depressed fracture on the skull, an antemortem fracture on the fifth metatarsal, and unmistakable signs of having been submitted to autopsy.

Considering the severity of the impact on the skull, we believe that this was almost certainly the cause of death of the individual. However, the forensic report claimed that the cause of death was a stroke — a consequence of atherosclerosis and alcoholism — and the report also mentions large amounts of fat, alluding to the mayor’s obesity. Taking this into consideration, we could conclude that the purpose of the autopsy report was not only to cover up torture and brutal murder, but also to defame the deceased by accusing him of poor health and “immoral” habits such as obesity and alcoholism.

Falsifying official forensic documents was common practice, as examples from other sources amply demonstrate. From the very beginning of the dictatorship, the opinions of forensic experts served to justify and whitewash brutal killings and torture. These irregularities in forensic practice, and their connection with the political repression of the period, require extensive historical and anthropological research to identify their occurrence.

Cases like this show the importance and necessity of the exhumation of mass graves and the anthropological analysis of those individuals buried in them, not only for the purpose of identifying the deceased, but also for exposing the truth recorded in the bodies of the victims and to gain a better understanding of the nature of political violence and the mechanisms of a fascist dictatorship.

## Key points

1. Military authorities during Franco’s dictatorship executed political opponents and buried them in mass graves.
2. The remains of individual identified as the last Republican mayor of Calera y Chozas had marks of autopsy, skull fracture and signs of torture.
3. According to the autopsy report the cause of death was a stroke.
4. The size and severity of the skull fracture point that the real cause of death was interpersonal violence.
5. This case is a clear example of the role of forensic medicine performed at the beginning of Franco's dictatorship.

## Data Availability

The datasets generated during and/or analyzed during the current study are available from the corresponding author on reasonable request.

## Footnotes

1 *BOE-*A-2011–15,206.

2 Autopsy report of the Military Court of Calera y Chozas, September 2, 1939, provided by the historian Javier de la Puerta.
